# Exposure to welding fumes increases lung cancer risk among light smokers but not among heavy smokers: evidence from two case–control studies in Montreal

**DOI:** 10.1002/cam4.6

**Published:** 2012-06-07

**Authors:** Eric Vallières, Javier Pintos, Jérôme Lavoué, Marie-Élise Parent, Bernard Rachet, Jack Siemiatycki

**Affiliations:** 1 CHUM Research Center, University of Montreal, MontrealQuebec, Canada; 2 INRS – Armand-Frappier Institute, University of QuebecLaval, Quebec, Canada; 3 London School of Hygiene and Tropical MedicineLondon, UK; 4 Department of Social and Preventive Medicine, University of MontrealMontreal, Quebec, Canada; 5 Guzzo-Cancer Research Society Chair in Environment and Cancer, School of Public Health, University of Montreal, MontrealQuebec, Canada

**Keywords:** Case–control studies, epidemiology, lung cancer, smoking, welding fumes

## Abstract

We investigated relationships between occupational exposure to gas and arc welding fumes and the risk of lung cancer among workers exposed to these agents throughout the spectrum of industries. Two population-based case–control studies were conducted in Montreal. Study I (1979–1986) included 857 cases and 1066 controls, and Study II (1996–2001) comprised 736 cases and 894 controls. Detailed job histories were obtained by interview and evaluated by an expert team of chemist–hygienists to estimate degree of exposure to approximately 300 substances for each job. Gas and arc welding fumes were among the agents evaluated. We estimated odds ratios (ORs) and 95% confidence intervals (CIs) of lung cancer using logistic regression, adjusting for smoking history and other covariates. The two studies provided similar results, so a pooled analysis was conducted. Among all subjects, no significant association was found between lung cancer and gas welding fumes (OR = 1.1; 95% CI = 0.9–1.4) or arc welding fumes (OR = 1.0; 95% CI = 0.8–1.2). However, when restricting attention to light smokers, there was an increased risk of lung cancer in relation to gas welding fumes (OR = 2.9; 95% CI = 1.7–4.8) and arc welding fumes (OR = 2.3; 95% CI = 1.3–3.8), with even higher OR estimates among workers with the highest cumulative exposures. In conclusion, there was no detectable excess risk of lung cancer due to welding fumes among moderate to heavy smokers; but among light smokers we found an excess risk related to both types of welding fumes.

## Introduction

Each year there are over one million deaths due to lung cancer, making this the most lethal malignancy worldwide [1]. Although tobacco smoking is the main determinant of lung cancer, accounting for 75–90% of incident cases, there is still an important fraction attributable to environmental and occupational exposures [2]. Identification of such agents is important for cancer prevention and compensation.

There has long been concern about the possible carcinogenicity of welding fumes [3, 4]. The term “welding fumes” refers to any fumes generated during the joining or cutting of metals using welding techniques [3]. There are a variety of welding techniques, the most common being arc welding (e.g., shielded metal arc welding, tungsten inert gas welding), where an arc between the filler metal and the work is the source of heat, and gas welding, where energy is provided by the combustion of oxygen and a gas such as acetylene. In addition to the welding technique, the composition of the fumes depends on the nature of base and filler metals, of filler fluxes, combustible, electrodes, and electrode coverings [3, 5, 6].

Previous epidemiological evidence linking exposure to welding fumes and lung cancer, some dating back several decades, presents inconsistent results. The early studies, mainly cohort studies of welders, showed an increase in risk, albeit not always statistically significant [7, 8]. More recent studies, including some case–control studies, focused on specific characteristics of welding fumes, like gas and arc welding fumes [9, 10], shipyard welding [11, 12], stainless steel or mild steel welding [4, 11, 12], and showed varied results without a clear underlying pattern. Since asbestos has been widely used in welding operations, its presence could confound the association with welding fumes [13, 14]. Furthermore, since many of the studies were retrospective cohort studies, they usually did not have access to complete lifetime smoking histories of study subjects. In 1990, the International Agency for Research on Cancer (IARC) categorized welding fumes in Group 2B – possibly carcinogenic to humans – based on limited evidence in humans and inadequate evidence in experimental animals.

In the early 1980s, we conducted a population-based case–control study in Montreal, Canada, to explore possible associations between hundreds of occupational substances and multiple cancer sites, including lung cancer. In the late 1990s, we carried out a similar study in the same area, this time focusing only on lung cancer. The purpose of these studies was to examine the effect of different occupational exposures at varying concentrations, and in a wide range of occupations. The aim of the present article is to investigate the risk of developing lung cancer associated with occupational exposure to gas and arc welding fumes, while adjusting for smoking history and other relevant covariates.

## Materials and Methods

Both studies entailed a case–control design and were based on the population of greater Montreal, numbering 2.7 million in 1981. The first study, labeled herein as Study I, was conducted between 1979 and 1986 and included men aged 35–70 and diagnosed with cancer at any of 19 sites [15, 16]. The second study, labeled as Study II, was conducted between 1996 and 2001 and included both men and women aged 35–75 diagnosed with a lung malignancy. Both studies included patients with incident, histologically confirmed tumors identified across all major Montreal area hospitals. Canada has a universal national health system, with no financial obstacles to physician or hospital services. For a disease as serious as lung cancer, it is most unlikely that any case would go undiagnosed. Every large diagnostic facility in the area participated in our study. Based on tabulations compiled for us by the Quebec Tumor Registry, we estimated that the small nonparticipating hospitals comprised no more than 5% of all lung cancer cases in Montreal. Both studies also included a series of population controls randomly selected from electoral lists. In the province of Quebec, electoral lists were maintained by means of active enumeration of households until 1994; they have since then been continually updated and are thought to represent nearly complete listings of Canadian citizens in the province. In both studies, population controls were frequency matched by age and area of residence (electoral district of about 40,000 individuals) to all cancer cases. Eligibility was restricted to Canadian citizens, resident in the greater Montreal area. Additional details of subject ascertainment and data collection have been presented previously [16, 17]. Results are presented herein for men only, because the prevalence of occupational exposure to welding fumes among women was very low in our study population (1%).

In Study I, 1082 lung cancer cases, 3634 cases of other types of cancer, and 740 population controls were identified and attempts were made to interview them. Of these, 857 (79%) lung cancer cases, 2896 (80%) other cancer cases, and 533 (72%) population controls completed the interview. To derive risk estimates in relation to lung cancer, we were able to use not only the population controls, but also a set of controls constituted from patients with other types of cancer. From the pool of other cancer patients, we selected a set of controls comprising 1349 cancer patients who had been ascertained in the same year and hospitals as the lung cancer cases, and selected so that none of the 19 individual cancer sites represented more than 20% of the overall pool of cancer controls. The main cancer sites in the cancer control series were colorectum (20%), bladder (17%), prostate (15%), stomach (9%), lymphomas (7%), and kidney (6%). There are different pros and cons associated with population controls and cancer controls [15, 18]. Although a population-based control group is often considered to be more representative of the base population, cancer controls are less susceptible to response bias and information bias [17]. We cannot affirm that one group is necessarily more valid than the other in representing the exposure experience of the study base. To enhance the power and to give equal weight to each type of control, we created a combined control group comprising all 533 population controls plus a random sample of 533 cancer controls, but only after verifying that the two groups gave similar risk estimates. The use of a pooled group equally weighted to population and cancer controls provides some protection against possible bias unique to each type of control group.

In Study II, 860 eligible male cases and 1294 eligible male controls were identified, and 736 (86%) and 894 (69%) of these, respectively, agreed to participate and satisfactorily completed the interview. Ethical approval was obtained for both studies from each participating hospital and university. All participating subjects provided informed consent before the interview.

In Study I, analyses using population and cancer controls were initially conducted separately. Since point estimates and confidence intervals (CIs) were similar using different control groups, we created a combined group comprising all 533 population controls and a random sample of 533 cancer controls.

### Data collection and exposure assessment

In Study I and Study II, over 78% and 76% of participants, respectively, responded for themselves, whereas proxy respondents provided information for the other participants. Interviews were divided into two parts: a structured section requested information on socio-demographic and lifestyle characteristics, including ethnicity, residential history, and smoking history, and a semi-structured section elicited a detailed description of each job held during the subject's working career.

For each job held, a trained interviewer asked the subject about the company, its products, the nature of the worksite, the subject's main and subsidiary tasks, and any additional information (e.g., equipment maintenance, use of protective equipment, activities of coworkers) that could provide clues about work exposures and their intensity. Occupations were coded according to the 1971 Canadian Classification and Dictionary of Occupations [19]. For some occupations, including welding, supplementary questionnaires were developed to assist interviewers with detailed technical probing, including questions about the type of gases used, metal welded, and the number of hours per week and weeks per year of exposure [20]. Our team of chemists and industrial hygienists, numbering from three to five at different times, examined each completed questionnaire and translated the description of each job into a list of potential exposures using a checklist of 294 agents that included gas and arc welding fumes. To accomplish this task the experts had access to a wealth of accumulated information and expertise on different industrial processes, some from published literature and some from local sources. The final exposure codes attributed to a participant were based on consensus among the coders. For each substance considered present in each job, the coders noted three dimensions of information, each on a three-point scale: their degree of confidence that the exposure had actually occurred (possible, probable, definite), the frequency of exposure in a normal workweek (<5%, 5–30%, >30% of the time), and the relative level of concentration of the agent (low, medium, high). Nonexposure was interpreted as exposure up to the level that can be found in the general environment. Exposure assessment was based not only on the worker's occupation, industry, and job title but also on individual characteristics of the workplace and tasks reported by the subject during the interview. Subjects with the same job title could have been attributed different exposure profiles, and conversely, similar exposures could be attributed to subjects with different job titles. An illustrative example can be found in the appendix of Parent et al. [21]. Coders were blind with regard to the subject's case or control status. Combining the two studies, more than 28,000 jobs were evaluated. The team of coders spent about 40 person-years on this project, which included developing the methodology, monitoring quality of interviewing, conducting background research on exposures in different occupations, coding the individual participants' files, and recoding after the initial complete round of coding was finished. A more extensive description of the exposure assessment method can be found elsewhere [[15–17, 22]].

### Data analysis

Unconditional logistic regression was used to estimate odds ratios (ORs) of lung cancer and 95% CIs for gas and arc welding fumes, while adjusting for several potential confounders as explained below. Subjects were categorized as unexposed or ever exposed to each type of welding fumes: those few subjects exposed only in the 5-year period prior to recruitment were considered unexposed. Ever-exposed individuals were further classified into nonsubstantial and substantial exposure subcategories. Substantial exposure was defined as exposure to medium or high fume concentrations for more than 5% of the work week, and for at least 5 years. Nonsubstantial exposure was assigned to the remaining exposed subjects.

The following nonoccupational covariates were included in all models: age, socioeconomic status (SES) measured by mean family income of the census tract of residence, education level, ethno-cultural origin (French, Anglo, others), respondent status (self, proxy), and tobacco smoking. Smoking was modeled as a combination of three dimensions (ever smoking status, cigarette-years, and time since quitting) based on a risk model derived from our study subjects proven to most accurately fit the data and combined into a comprehensive smoking index (CSI) [23, 24]. The CSI is correlated with cumulative cigarette-years, but not perfectly, since it takes into account the timing of smoking exposure, and not just the duration and intensity. Our dataset included estimates of exposure to many occupational exposures, some of which are recognized lung carcinogens, namely asbestos, nickel, chromium VI, cadmium, and arsenic. We considered including these in the statistical models as potential confounders. Arsenic was excluded because of its very low prevalence in our population. Nickel, chromium VI, and cadmium were excluded because these substances are inherent components of welding fumes rather than being correlated but distinct exposures. Only asbestos, a frequent coexposure, but not inherently part of welding fumes, was retained as a covariate to include in the models; it was simply entered as a binary (present/absent) variable. Sensitivity analyses were carried out with several other suspected lung carcinogens in the models.

In addition to analyzing Study I and Study II separately, we tested for heterogeneity of results between studies, and when warranted, carried out analyses on the pooled datasets; for this, we added to the models a variable for the study (I or II).

Besides treating smoking as an a priori confounder, we explored potential effect-measure modification by smoking. Since the number of never smokers among cases was very low, the nonsmokers category was supplemented with lifetime low intensity smokers. Operationally we defined lifetime low intensity smokers as individuals having a CSI value below the 25th percentile on this scale. Because of the way it is constructed, the CSI index does not translate simply onto the duration or daily amount of pack-year scale. We can illustrate the amount of smoking in these categories by showing two smoking profiles that would fall on the 25th percentile of the CSI scale, namely: a current smoker who smoked three cigarettes per day during 40 years, (with a lifetime cumulative exposure of 6 pack-years), or a former smoker who smoked six cigarettes a day for 30 years and quit 10 years ago (and has a cumulative exposure of 9.8 pack-years). Smokers whose CSI value was above the 25th percentile were considered medium/heavy smokers. To evaluate the statistical significance of the difference in ORs between the two strata of smokers, we carried out an analysis in which all subjects were included, and the two variables, smoking status (binary) and exposure to welding fumes (binary), were included and their cross-product term was tested. This was repeated for each type and level of welding fumes. When analyzing the effects of welding fumes in separate strata of smoking, we still included the continuous CSI variables as a covariate in the models, to avoid residual confounding within the smoking status strata.

The associations between welding fumes and the most prevalent histologic types of lung cancer, namely squamous cell, adenocarcinoma, and small cell, were also evaluated.

## Results

Table 1 shows the distribution of cases and controls according to different socio-demographic characteristics. In both studies, compared to controls, cases were more often of French ancestry, had fewer years of formal education, lived in census tract regions with lower mean family income, and interviews were more likely to have been conducted with a proxy respondent than controls. As expected, cases were also more likely to be current smokers and had smoked more intensely than controls. There is a significant decrease in the proportion of current smokers between the two studies, particularly among the controls, in conformity with national statistics indicating that smoking rates declined among males in the late 20th century [25].

**Table 1:** Selected socio-demographic characteristics of male subjects

		Study I (1979–1986)			Study II (1996–2001)	
			
Variables	Categories	Population controls (*N* = 533)	Cancer controls (*N* = 1349)	Cancer cases (*N* = 857)	Population controls (*N* = 894)	Cancer cases (*N* = 736)
Age group (%)	≤55	28.0	32.5	27.4	11.9	13.6
	56–65	45.2	43.7	50.8	28.6	32.9
	66–75	26.8	23.7	21.8	59.5	53.5
Ethno-linguistic group (%)	French	64.2	58.0	69.1	64.4	77.4
	English	14.1	16.1	13.5	6.4	4.6
	Other	21.8	25.9	17.4	29.2	17.9
Schooling (%)	<7 years	20.3	22.3	30.3	24.7	28.0
	7–12 years	56.1	55.2	57.1	48.1	56.3
	≥13 years	23.6	22.5	12.6	27.2	15.7
Median family income*		100	93	84	100	94
Smoking (%)	*N*ever	19.7	17.3	1.5	17.7	2.4
	Current	46.9	58.0	79.9	29.2	67.5
Quit smoking (%)	2–5 years ago	8.8	6.7	7.6	2.8	4.3
	5–10 years ago	7.9	6.2	6.0	6.6	5.8
	>10 years ago	16.7	11.8	5.0	43.7	19.8
Mean pack-years**		49.9	52.3	74.3	50.3	78.5
Respondent (%)	Self	87.4	80.8	70.6	90.3	60.2
	Proxy	12.6	19.2	29.4	9.7	39.8

* Indicator of intersubject mean of the median family income for census tract of residence, using the study-specific mean value among population controls as the reference value for each study (×100). Based on the 1981 census for Study I and the 1991 census for Study II.

** Among ever smokers, based on 20 cigarettes per packet.

Table 2 shows the lifetime prevalence of occupational exposure to both gas and arc welding fumes, for all participants in the two case–control studies. Lifetime occupational ever exposure to welding fumes ranged from 11 to 18%. At the substantial level of exposure, the prevalence of exposure to gas and arc welding fumes was approximately 5%, with a slightly higher percentage for cases, compared to controls. The proportion of subjects with lifetime exposure to both gas and arc welding fumes did not differ much by study or by disease status.

**Table 2:** Distribution of male subjects according to lifetime occupational exposure to gas and arc welding fumes

	Study I (1979–1986)						Study II (1996–2001)			
		
	Population controls (*N* = 533)		Cancer controls (*N* = 1349)		Cancer cases (*N* = 857)		Population controls (*N* = 894)		Cancer cases (*N* = 736)	
					
	*n*	%	*n*	%	*n*	%	*n*	%	*n*	%
*Gas welding fumes*										
Never exposed	477	89.5	1196	88.7	742	86.6	742	83.0	627	85.2
Ever exposed	56	10.5	153	11.3	115	13.4	152	17.0	109	14.8
Nonsubstantial level	33	6.2	82	6.0	62	7.2	120	13.4	79	10.7
Substantial level	23	4.3	71	5.3	53	6.2	32	3.6	30	4.1
*Arc welding fumes*										
Never exposed	461	86.5	1193	88.5	752	87.7	727	81.3	622	84.5
Ever exposed	72	13.5	156	11.5	105	12.3	167	18.7	114	15.5
Nonsubstantial level	50	9.4	99	7.3	61	7.1	131	14.7	81	11.0
Substantial level	22	4.1	57	4.2	44	5.2	36	4.0	33	4.5
*Gas and arc welding fumes*										
Never exposed to either	442	82.9	1137	84.3	701	81.8	689	77.1	589	80.0
Only gas welding fumes	19	3.6	54	4.0	50	5.8	38	4.3	33	4.5
Only arc welding fumes	35	6.6	59	4.4	41	4.8	53	5.9	38	5.2
Both gas and arc fumes	37	6.9	99	7.3	65	7.6	114	12.8	76	10.3

During the period of greatest relevance of this study (1945–1996), the industrial profile of the Montreal area was quite varied and there was considerable heavy industrial activity. Some of the major industries in the area, in which welding activities are prevalent, were: rail transportation companies, aircraft manufacturing industries, shipyards, iron and steel foundries, and industries manufacturing electric, electronic, and telecommunication products. Table 3 presents the occupations most commonly exposed to gas and arc welding fumes in our two study sample. The top four categories on the list, welders and flame cutters, motor-vehicle mechanics, pipe-fitters and plumbers, and machinery mechanics, account for one-third of the total number of jobs entailing welding fume exposure.

**Table 3:** The main occupations held by male subjects exposed to gas and arc welding fumes

		Gas welding fumes		Arc welding fumes	
			
CCDO* code	Occupation title	Study I (*n* = 465) (%)***	Study II (*n* = 442) (%)	Study I (*n* = 516) (%)	Study II (*n* = 518) (%)
8581	Motor-vehicle mechanics and repairers	12.7	17.4	5.4	11.2
8335	Welding and flame cutting occupations	14.8	13.8	20.3	15.1
8791	Pipefitting, plumbing, and related occupations	6.7	3.6	3.1	4.6
8584	Machinery mechanics and repairers	5.6	4.1	6.8	2.9
8313	Machinist and machine-tool setting-up occupations	3.7	2.9	4.8	2.3
8580	Foremen/women, mechanics and repairers, except electrical	3.0	3.2	2.1	2.5
8799	Other construction trades occupations	2.2	3.4	1.4	2.5
8333	Sheet-metal workers	3.2	1.8	3.9	3.1
8583	Rail transport equipment mechanics and repairers	2.8	2.0	2.1	1.0
8793	Structural-metal erectors	1.7	1.6	1.4	2.3
8393	Filing, grinding, buffing, cleaning and polishing occupations	1.3	1.6	1.7	1.7
8533	Electrical and related equipment installing and repairing occupations	0.9	2.0	1.4	1.7
8515	Aircraft fabricating and assembling occupations	2.2	0.5	2.7	1.2
9311	Hoisting occupations	1.7	0.9	1.4	1.7
5130	Supervisors: sales occupations, commodities	1.7	0.7	0.8	0.0
8591	Jewelry and silverware fabricating and repairing	1.1	1.4	0.0	0.0
	All other jobs with this exposure	34.8	39.1	40.7	46.1

* CCDO, Canadian Classification and Dictionary of Occupations.

**Numbers of jobs with exposure to each compound. Each subject may have been exposed in more than one job.

*** Percentage of subjects with this type of welding exposure who were in each listed occupation.

In Study I, the ORs between welding fumes and lung cancer were very similar whether using population controls or cancer controls, so we used the combined group of controls for all the remaining analyses of Study I. Table 4 shows the adjusted ORs for the relationship between lung cancer and occupational exposures to gas and arc welding fumes, for each study separately and for the pooled dataset from both studies. Overall, risk estimates were higher in Study I than in Study II, but overall patterns were similar. In Study I, we found a small increased risk of lung cancer for any level of exposure to gas welding fumes only (OR = 1.4; 95% CI = 1.0–2.0). None of the OR estimates for Study II reached statistical significance. The pooled analysis did not show an increased risk of lung cancer after exposure to gas welding fumes (OR = 1.1; 95% CI = 0.9–1.4), nor to arc welding fumes (OR = 1.0; 95% CI = 0.8–1.2). No dose–response pattern was found between exposures at a substantial level versus a nonsubstantial level. The numbers exposed at the substantial level were small, so risk estimates for this category were rather unstable. We also computed ORs separately for the different dimensions of cumulative exposure, such as duration (Table 4), frequency, and concentration (data not shown). None of these dimensions showed clear trends for either type of welding fumes.

**Table 4:** Odds ratio of lung cancer associated with occupational exposure to gas and arc welding fumes among Montreal males in two studies and a pooled analysis

	Study I (1979–1986)				Study II (1996–2001)				Pooled studies**			
			
	Controls (*n*)	Cases (*n*)	OR*	95% CI	Controls (*n*)	Cases (*n*)	OR*	95% CI	Controls (*n*)	Cases (*n*)	OR	95% CI
*Gas welding fumes*												
Nonexposed	949	742	1.0	–	742	627	1.0	–	1691	1369	1.0	–
Any level of exposure	116	115	1.4	1.0–2.0	152	109	0.9	0.6–1.2	268	224	1.1	0.9–1.4
Any level ≤20 years	61	68	1.7	1.1–2.6	86	68	1.0	0.7–1.5	147	136	1.3	1.0–1.7
Any level >20 years	55	47	1.1	0.7–1.8	66	41	0.7	0.4–1.2	121	88	0.9	0.7–1.3
Nonsubstantial level	65	62	1.5	1.0–2.3	120	79	0.9	0.6–1.3	185	141	1.1	0.9–1.5
Substantial level	51	53	1.3	0.8–2.1	32	30	0.8	0.5–1.5	83	83	1.1	0.8–1.6
*Arc welding fumes*												
Nonexposed	931	751	1.0	–	727	622	1.0	–	1658	1373	1.0	–
Any level of exposure	135	106	1.0	0.7–1.3	167	114	1.0	0.8–1.4	302	220	1.0	0.8–1.2
Any level ≤20 years	77	63	1.0	0.7–1.5	102	73	1.1	0.7–1.6	179	136	1.1	0.8–1.4
Any level >20 years	57	43	0.9	0.6–1.4	65	41	0.9	0.6–1.5	122	84	0.9	0.6–1.3
Nonsubstantial level	90	62	0.9	0.6–1.3	131	81	1.0	0.7–1.4	221	143	0.9	0.7–1.2
Substantial level	45	44	1.1	0.7–1.8	36	33	1.2	0.7–2.1	81	77	1.1	0.8–1.6

* Adjusted for age, ethno-linguistic group, years of education, respondent status, cigarette index, and asbestos exposure.

** Adjusted for same variables, plus indicator for study.

Several sensitivity analyses were conducted. One set of analyses was conducted among self-respondents only, excluding proxy respondents altogether. Another set was conducted only among subjects of French ancestry, thereby eliminating any possibility of residual confounding by ethnicity. In recognition of the fact that welding fumes sometimes includes certain metals and that these may induce some effect modification, we performed analyses of welding fumes with and without each of the following coexposures: nickel, cadmium, chromium VI, and stainless steel. Because these substances are inherent components of welding fumes rather than being correlated, we assessed different ways of defining these variables for analysis, namely we defined the following alternative exposure categories: welding fumes including metals, welding fumes without metals, and metal fumes without any welding fumes. None of these sensitivity analyses produced results that differed materially from those shown in Table 4 (data not shown).

Table 5 shows OR estimates for exposure to welding fumes, stratified by smoking status. For gas welding fumes (OR = 2.9; 95% CI = 1.7–4.8) and arc welding fumes (OR = 2.3; 95% CI = 1.3–3.8), we found a significantly increased risk of lung cancer due to welding fumes among non/low smokers, but not among moderate/heavy smokers. Point estimates among moderate/heavy smokers were close to null. When we further narrowed attention to non/low smoking workers with substantial exposure to fumes, we found even higher risks of lung cancer for gas (OR = 4.8; 95% CI = 2.2–10.4) and arc welding fumes (OR = 3.6; 95% CI = 1.6–7.8).

**Table 5:** Odds ratio of lung cancer associated with occupational exposure to gas and arc welding fumes among Montreal males in a pooled analysis, stratified by smoking status, and test for interaction

	Smoking status								
		
	Never–low smokers				Medium–heavy smokers				
			
	Controls (*n*)	Cases (*n*)	OR*	95% CI	Controls (*n*)	Cases (*n*)	OR*	95% CI	*P*–value**
*Gas welding fumes*									
Nonexposed	670	91	1.0	–	1022	1278	1.0	–	
Any level of exposure	93	33	2.8	1.7–4.8	176	191	0.9	0.7–1.2	0.000
Nonsubstantial level	72	18	2.3	1.2–4.2	113	123	1.0	0.7–1.3	0.005
Substantial level	21	15	4.3	1.9–9.7	63	68	0.8	0.6–1.2	0.000
*Arc welding fumes*									
Nonexposed	654	93	1.0	–	1005	1280	1.0	–	
Any level of exposure	109	31	2.2	1.3–3.7	193	189	0.9	0.7–1.1	0.000
Nonsubstantial level	83	18	1.7	0.9–3.2	138	125	0.8	0.6–1.1	0.009
Substantial level	25	13	3.5	1.6–7.8	55	64	0.9	0.6–1.3	0.002

* Adjusted for age, ethno-linguistic group, years of education, respondent status, cigarette index, asbestos exposure, and study.

** Significance of the interaction term between smoking (binary) and each type of welding fumes (binary), in the regression model.

To explore whether the apparent effect modification by smoking status may be related to different types of welding exposure circumstances or to different socio-demographic characteristics, we compared subjects in the two strata. Compared with medium/heavy smokers, non/low smokers were more often of non-French ethnicity and of relatively higher SES. Although the patterns of duration and concentration of exposure were similar, there were slightly more very long duration-exposed workers among the non/low smokers.

When the smoking strata were combined and an interaction term was tested between smoking status and welding fume exposure, the interaction terms were highly significant, indicating that the differences in risk estimates between moderate/heavy and non/low smokers were unlikely to be caused by chance (last column of Table 5). The pattern of OR results in Table 5 is closer to an additive than a multiplicative model between smoking and welding fumes.

We conducted analyses analogous to those reported for all lung cancers, but focusing separately on the three main histological types: squamous cell, small cell, and adenocarcinoma. Because of smaller numbers in these sub-site analyses, we present the results of the pooled data from Studies I and II. Table 6 shows the key findings from these analyses. In analyses of nonsmokers and smokers combined, the ORs are near null for all three histological types, but this again masks different patterns between non/low smokers and moderate/heavy smokers. Among non/low smokers, workers exposed to both types of welding fumes had high ORs for each of the three types of lung cancer, but the OR was strongest for squamous cell and weakest for adenocarcinoma.

**Table 6:** Odds ratio of lung cancer associated with occupational exposure to gas and arc welding fumes by histological types in a pooled analysis of Study I and II

		Squamous cell			Small cell			Adenocarcinoma		
				
	Controls (*n*)	Cases (*n*)	OR*	95% CI	Cases (*n*)	OR	95% CI	Cases (*n*)	OR	95% CI
*Gas welding fumes*										
All subjects										
Nonexposed	1691	528	1.0	–	237	1.0	–	356	1.0	–
Any level	268	92	1.1	0.8–1.5	47	1.3	0.9–1.9	52	1.0	0.7–1.4
Nonsubstantial level	185	61	1.2	0.8–1.7	28	1.3	0.8–2.1	33	1.0	0.6–1.5
Substantial level	83	31	1.0	0.6–1.6	19	1.3	0.7–2.3	19	1.0	0.6–1.8
Never/low smokers										
Nonexposed	670	25	1.0	–	17	1.0	–	26	1.0	–
Any level	92	13	4.8	2.1–10.9	6	2.9	0.9–9.2	7	1.5	0.6–4.1
Medium/heavy smokers										
Nonexposed	1022	503	1.0	–	220	1.0	–	330	1.0	–
Any level	176	79	0.9	0.7–1.2	41	1.2	0.8–1.8	45	0.9	0.6–1.4
*Arc welding fumes*										
All subjects										
Nonexposed	1658	523	1.0	–	245	1.0	–	353	1.0	–
Any level	302	97	1.1	0.8–1.5	39	0.9	0.6–1.4	55	1.0	0.7–1.4
Nonsubstantial level	221	64	1.1	0.8–1.5	26	1.0	0.6–1.6	33	0.9	0.6–1.3
Substantial level	81	33	1.3	0.8–2.1	13	0.9	0.5–1.6	22	1.2	0.7–2.1
Never/low smokers										
Nonexposed	654	24	1.0	–	18	1.0	–	26	1.0	–
Any level	108	14	4.5	2.0–10	5	2.1	0.6–7.0	7	1.4	0.5–3.7
Medium/heavy smokers										
Nonexposed	1005	499	1.0	–	227	1.0	–	327	1.0	–
Any level	193	83	0.9	0.7–1.3	34	0.9	0.6–1.3	48	0.9	0.6–1.3

* Adjusted for age, ethno-linguistic group, years of education, respondent status, cigarette index, asbestos exposure, and study.

## Discussion

Millions of workers are exposed to welding fumes worldwide. Although the prevalence and intensity of exposure may be declining in North America and Europe [26], it is likely that such industrial activities are relocating to developing countries, where regulation of the occupational environment tends to be less stringent.

Among the large variety of welding techniques, arc and gas welding fumes are the most prominent. Composition of fumes depends on several factors, such as the metal being welded, the type of electrode used, and the choice of shielding gas. Given the great variability, it is very difficult to identify all the components of welding fumes, and the role which each of these components plays in the etiology of lung cancer. In examining the patterns of coexposures attributed by our team of expert industrial hygiene raters, it was seen that stainless steel dusts, chromium VI, and nickel were very often attributed when there was arc welding. Aluminum and mild steel dust were also very prevalent with both gas and arc welding fumes.

### Contextual and methodological considerations

The potential carcinogenic effect of welding fumes was investigated in our study in a wide variety of jobs and industries, and often where welding was not necessarily the primary task of the worker. Among those jobs coded as “welders and flame-cutters,” which represents 15–20% of the jobs that entailed welding fume exposure in our study sample, the median frequency of welding activity was around 38 h/week and the concentration was usually coded as high; among the remaining 80–85% of jobs with welding fume exposures, the median frequency was 5 h/week, with a low or medium concentration. This is in contrast with most previous cohort studies where the study populations were selected for study because of their intense welding activities. Thus, on one hand, it is likely that the workers exposed to welding fumes in our study were exposed on average less intensively than workers in previous cohort studies of welders, and it is possible that the exposure levels were insufficient to induce a risk that might be detectable at higher levels. On the other hand, the distribution of exposure circumstances in our study population is probably more representative of exposure circumstances to welding fumes across the industrial spectrum. Our studies also allowed for the integration of lifetime job histories, rather than focusing on the worker's history with only one employer.

Occupational exposure was attributed to subjects on the basis of their detailed lifetime job history reported at the interview and assessment by a team of exposure experts. We have previously shown that subjects' reports of occupational history were valid [27], and that our team of chemists and industrial hygienists attributed exposure with reasonable reliability [28] and validity [29]. Nevertheless, the retrospective exposure assessment procedure was not based on active measurement (an impossibility in retrospective case–control studies) and therefore entailed some degree of measurement error. Concentration of exposure could not be estimated in absolute terms; it was only done on an ordinal scale. Because this work was done blindly with respect to disease status, any misclassification of exposure would have occurred nondifferentially with respect to outcome.

The number of subjects were large for a population-based case–control study with detailed exposure assessment; nevertheless the various OR estimates were not very precise. There may have been selection biases if workers who were particularly susceptible to lung cancer and other respiratory diseases could have selected themselves out of welding occupations after relatively brief employment because they suffered adverse short-term respiratory effects from the dusty conditions. Response rates were quite high, over 80% for case groups and 70% for population control groups. These proportions were as high as or higher than those in most recent studies [30], thus diminishing the likelihood of nonparticipation bias. Adjustment for potential confounders was more extensive than in previous studies. We collected detailed information on potential confounders, covering socio-demographic and lifestyle factors including detailed smoking history, as well as other occupational exposures pertinent to lung cancer such as asbestos, chromium VI, arsenic, and cadmium. Whereas it is notoriously difficult to control for smoking in retrospective cohort studies, a case–control study allows for ascertainment of complete history of cigarette smoking. For the parameterization of smoking history variables, we used an approach based on a risk model derived from our study subjects [23]. The case definition of incident histologically confirmed lung cancer allowed for the collection of more detailed diagnostic information from medical records than that typically found on death certificates. There were quite high proportions of proxy response, but it was reassuring that the prevalence of exposure was similar between proxy and self-respondents, and that the overall results were similar when we excluded subjects with proxy respondents from the analyses (data not shown). For all these reasons, results from our case–control studies constitute an important complement to previous studies.

### Risk results

Several epidemiological studies reported a 30–40% excess risk of lung cancer for workers exposed to welding fumes [[4, 7, 9, 31–41]]. These results are difficult to compare between each other given the likely large interstudy differences in the type of fumes, intensity of exposure, and the ability to control for the main potential confounders, like tobacco smoking, asbestos, and SES. Nor is there consistency of findings among those studies that purported to adjust for these confounders. Some authors conclude that the apparent risk related to welding fumes is actually attributable to asbestos or tobacco smoking [9, 10, 42, 43], while others indicate that this excess risk cannot be explained only by these confounders [4, 12, 34, 44].

Most men in our study population were smokers, and the main results (Table 4) on welding fumes can be interpreted essentially as estimates of the risk of lung cancer due to welding fumes in a population of smokers. Apart from a hint of excess risk for those exposed to gas welding fumes in Study I, we did not detect any excess risk. Among those few previous studies that made a distinction between gas and arc welding, two showed an elevated risk after exposure to arc welding fumes only [9, 45]. We failed to find a trend with increasing concentration and/or duration of exposure, unlike some other studies [4, 31, 45, 46]. In general these findings were compatible with those of most previous studies, where the estimates of relative risk have ranged from null to slightly elevated.

It was when we stratified the study sample by smoking status, and in particular when we examined the welding effects among non/low smokers, that the association of welding fumes, both gas and arc, with lung cancer became quite strong and clear, with indications of dose–response. It would be helpful to examine these associations in “pure” lifetime nonsmokers, but there were not enough of them in our study (1.5% in Study I and 2.4% in Study II) to support statistical analyses. Since we included some low level smokers in the non/low stratum, it is conceivable that there was some residual confounding by smoking even among the non/low smokers. But the inclusion of the CSI variable in the intrastratum analyses likely took care of any detectable residual confounding. To our knowledge, there are no previous investigations showing such a stark effect modification. This heterogeneity of results between smoking strata might be explained by the strong effect of tobacco smoking, which could mask the relatively small effect of gas and arc welding fumes on lung cancer, especially among the heavy smoking population in our study. In our study population, we observed a strong effect of smoking history on lung cancer risk, with a clear dose–response relationship. Combining the two studies, the estimated OR between ever smoking and lung cancer was 2.2 (95% CI = 1.4–3.6) among smokers with less than 20 pack-years of cumulated smoking, and that among subjects with >20 pack-years was 11.4 (95% CI = 7.8–16.7).

There have been few previous studies of risks of welding fumes for different histological types of lung cancer. An Argentine hospital-based study reported a strong, yet imprecise excess risk of squamous cell carcinoma among workers exposed to welding fumes [47], while a French hospital-based study reported a significant excess risk of adenocarcinoma among workers exposed to welding fumes [48]. Our patterns of results by histological type of lung cancer were similar to our results for all lung cancers combined – namely no apparent excess risks among all subjects or among medium/heavy smokers, but some indication of excess risk among non/low smokers. Those possible excess risks among non/low smokers varied by histological types: the strongest associations were with squamous cell tumors and the weakest with adenocarcinomas. This pattern of findings is compatible with several interpretations. Since the association between smoking and lung cancer is strongest for squamous and small cell cancer and weakest for adenocarcinoma [49, 50], it may be that the pattern of results we observed represents residual confounding by smoking, despite our intensive efforts to adjust for smoking. It is not clear why such confounding would operate only in the non/low stratum of smokers, but it remains hypothetically possible. Another interpretation, more likely in our view, is that the carcinogenic effect of welding fumes operates through a similar mechanism as the effect of smoking and that the pattern of histological results reflects this shared mechanism. Arc welding produces finer fume particles than gas welding, due to high temperature oxidation. About 90% of arc welding fume particles are smaller than 2 μm and therefore can penetrate deeper in the lower respiratory tract [5]. We would have expected to find a stronger association between arc welding and adenocarcinoma (which is usually peripheral lung cancer) than for gas welding and other cell types, but this did not transpire in our study.

In summary, the results of our study do not show a clear and increased risk of lung cancer linked to occupational exposure to gas and/or arc welding fumes among medium/heavy smokers, constituting about 75% of our study subjects. However, our results indicate an increased risk due to both gas and arc welding fumes among never and mild smokers, and the risks are higher among those subjects with higher cumulative exposure.
